# Blood DNA Methylation Responses to Seasonal Environmental Variation in the Giant Panda (*Ailuropoda melanoleuca*): A Whole-Genome Bisulfite Sequencing Analysis of Multiple Individuals Across Multiple Time Points

**DOI:** 10.3390/ani16203286

**Published:** 2026-10-18

**Authors:** Jiabin Liu, Rui Ma, Wenlei Bi, Jiang Gu, Jiaojiao Yu, Xiang Yu, Long Zhang, Chong Huang, Zusheng Li, Feifei Feng, Fei Xue, Guanwei Lan, Yanshan Zhou, Chao Chen, Daode Yang, Dunwu Qi

**Affiliations:** 1 Institute of Wildlife Conservation, Central South University of Forestry and Technology, Changsha 410004, China; jiabin_liu2013@126.com; 2 The Conservation of Endangered Wildlife Key Laboratory of Sichuan Province, Chengdu Research Base of Giant Panda Breeding, Chengdu 610081, China

**Keywords:** giant panda, DNA methylation, seasonal adaptation, whole-genome bisulfite sequencing, differentially methylated regions

## Abstract

Seasonal environmental variation is a widespread stressor for wildlife and can induce plastic changes in DNA methylation. Blood DNA methylation is an integrative molecular indicator of overall physiological status, yet its seasonal dynamics in the giant panda remain unclear. This study aimed to profile genome-wide blood methylation patterns in giant pandas under natural field conditions and to reveal the epigenetic strategies underlying their seasonal adaptation. Twelve blood samples were collected from three wild-trained giant pandas in the Daxiangling Nature Reserve during winter (*n* = 6) and summer (*n* = 6) between 2023 and 2025 and were subjected to whole-genome bisulfite sequencing. The genome-wide mC/C ratio (4.18–4.98%) was dominated by the CG context. Winter–summer comparison identified 7113 differentially methylated regions and 3063 differentially methylated genes. These genes were enriched in transcriptional regulation, cell surface receptor signaling, miRNA metabolism, and MAPK, Ras, and T/B cell receptor signaling pathways. Winter-hypermethylated genes were enriched in both immune defense and energy-consuming processes, whereas winter-hypomethylated genes showed limited enrichment, indicating that hypermethylation-mediated suppression dominates seasonal adaptation. This study reveals, for the first time, a seasonal methylation fingerprint in giant panda blood characterized by a “summer immune enhancement, winter energy conservation” pattern. As sampling occurred in natural habitats, these differences reflect integrated epigenetic responses to temperature, photoperiod, food availability, and pathogen pressure rather than single-factor effects. These findings provide molecular evidence for seasonal adaptation in endangered mammals and candidate markers for captive panda management and wild-release assessment.

## Simple Summary

The giant panda is a rare and endangered animal found only in China. It lives in mountains where conditions change dramatically between seasons: winters are cold and dry with little food, while summers are warm and humid, when ticks and other harmful small creatures become active. We aimed to explore how the panda’s body responds to these seasonal changes. We studied blood samples collected in winter and summer from three pandas living outdoors in a nature reserve. Inside every animal, tiny chemical “switches” attach to the genetic material and help decide which genes are turned on or off. We found that the pattern of these switches clearly differed between winter and summer. In summer, switches that boost immune defenses were turned on, helping the body to fight parasites; in winter, switches controlling energy-consuming activities were turned off, helping the animal to save energy. We call this the “summer immune enhancement, winter energy conservation” pattern. Understanding these hidden seasonal adjustments can help caretakers better protect pandas in zoos, support the health of pandas being prepared for release into the wild, and offer insights into how other endangered mammals cope with seasonal hardships in nature.

## 1. Introduction

Seasonal environmental variation is one of the most pervasive and complex environmental stressors confronting wild animals in their natural habitats [1]. As seasons change, photoperiod, ambient temperature, precipitation and humidity, food resource abundance and quality, and the activity of parasites and other pathogens undergo systematic and coordinated shifts, collectively shaping the seasonal environmental regimes of animal habitats [2]. To cope with such multifactorial seasonal pressures, animals have evolved sophisticated adaptive strategies across behavioral, physiological, and molecular levels [3,4,5,6].

Among wild animals affected by seasonal environmental stressors, the giant panda (*Ailuropoda melanoleuca*) is particularly representative. Giant panda habitats are characterized by distinct seasonality—cold, dry winters and warm, humid summers. During winter, low ambient temperatures and food scarcity impose stress on the maintenance of energy homeostasis [7,8], whereas the warm, humid summer climate facilitates the survival and transmission of ectoparasites and endoparasites [9,10,11], thereby compounding health risks at the immunological level. Indeed, tick burdens on giant pandas at Daxiangling Nature Reserve peak during summer [10,11], and similar warm-season peaks in infection intensity of the gastrointestinal ascarid *Baylisascaris schroederi* have been documented at Foping Nature Reserve [9]. Through long-term adaptive evolution, giant pandas exhibit pronounced seasonal variation in foraging behavior, feeding on different bamboo species and different parts of the same species (shoots, culms, and leaves) across seasons [12,13,14], which is closely associated with marked differences in protein, lipid, carbohydrate, and fiber content among bamboo species and plant parts [12,13]. GPS collar studies have further shown that their activity rhythms and energy budgets undergo systematic seasonal adjustments [3,15]. In addition, doubly labeled water studies have revealed that giant pandas adopt a relatively low energy expenditure strategy, with field metabolic rates lower than those predicted for mammals of comparable body size [7,16], and can actively upregulate metabolic rate to maintain body temperature under low winter temperatures [16]. However, the molecular regulatory mechanisms underlying these seasonal adaptive phenotypes, particularly the epigenetic response patterns, remain largely unknown.

DNA methylation is one of the most stable and best-studied epigenetic modifications in eukaryotes, and plays a central role in maintaining genomic stability, regulating gene expression, and mediating cellular differentiation [17,18,19,20,21,22,23]. As a key molecular hub linking environment, genotype, and phenotype, DNA methylation responds sensitively to diverse environmental stimuli [24,25,26,27,28]. With respect to temperature, cold exposure and heat stress can reshape DNA methylation profiles across species from mice, guinea pigs, and cattle to humans, involving multiple physiological dimensions such as energy metabolism and immune responses [29,30,31,32]; the hibernating thirteen-lined ground squirrel also exhibits specific methylation modifications of metabolism-related genes under natural hypometabolic states [33]; even in fish, short-term temperature fluctuations (e.g., cold stress in zebrafish and 2 °C warming in European sea bass) are sufficient to induce substantial methylation remodeling [34,35,36]. With respect to photoperiod, day length regulates mammalian seasonal physiological rhythms via hypothalamic methylation [37,38], and seasonal methylation variation in wild birds is linked to breeding phenology [4,39]. With respect to pathogens, infection can induce host epigenetic modifications, including DNA methylation, to modulate immune responses, whereas pathogens themselves may actively target the host methylome to suppress immunity and achieve immune evasion [40]. Collectively, these cross-species findings indicate that DNA methylation is a highly sensitive responder to seasonal environmental variation and that the adaptive mechanisms it mediates are evolutionarily conserved from fish to mammals [4,24,25,29,31,33,34,35,37,38,39,41]. However, most of this evidence derives from controlled experiments or model and economically important animals, and direct evidence of seasonal epigenetic responses in large wild mammals inhabiting natural habitats remains extremely scarce.

As an important representative of endangered species, DNA methylation research in the giant panda has achieved preliminary progress. Single-base-resolution methylation maps have been generated for brain, liver, and pancreas tissues [42,43], revealing the regulatory roles of DNA methylation in tissue development and differentiation. Comparative methylation analysis has also been performed on blood samples from captive elderly pandas with cataracts and healthy individuals [44], providing the first evidence that DNA methylation contributes to age-related diseases in this species. In addition, an age-related dynamic blood DNA methylation profile has been constructed using whole-genome bisulfite sequencing (WGBS) [45], revealing a genome-wide decline in methylation levels with age and methylation–expression correlations in immune- and metabolism-related genes. Nevertheless, studies investigating the epigenetic responses of wild animals—especially endangered large mammals—to seasonal environmental variation in natural habitats remain extremely limited. Giant pandas in the wild must simultaneously contend with elevated pathogen pressure (e.g., ticks) driven by the hot and rainy summer [10], as well as food scarcity and heightened energy conservation demands imposed by cold winters [8]. Such seasonal differences in survival pressures may leave detectable imprints on their blood DNA methylome. Wild training refers to the practice of raising captive-bred giant pandas in large, semi-natural enclosures that simulate field conditions, whereby the animals progressively develop the survival competence required for independent life in the wild. Because repeated blood sampling from fully wild giant pandas is not feasible, wild-trained individuals living under such near-natural conditions represent the most practical surrogate for investigating seasonal DNA methylation dynamics under field conditions. This population thus serves a dual purpose: assessing captive individual adaptation during the wild training process, and providing, by proxy, insight into the seasonal epigenetic responses of wild pandas that cannot be directly sampled. Although a previous study characterized the blood DNA methylation profiles of wild-trained giant pandas [46], the patterns by which seasonal environmental variation drives alterations in the giant panda blood methylome, and their potential functional significance, remain entirely unknown.

Because blood circulates throughout the body and is functionally coupled with immune, endocrine, and metabolic systems, blood DNA methylation provides a molecular readout of the organism’s overall physiological state [25,32,45,47]. Given that other tissues cannot be sampled repeatedly from giant pandas, blood further represents the only feasible matrix for longitudinal epigenetic monitoring in this species. Accordingly, this study focused on wild-trained giant pandas living under natural field conditions in the Daxiangling Nature Reserve. Using WGBS, we performed single-base-resolution methylome analyses on blood samples collected in summer and winter over three consecutive years, aiming to: (1) systematically characterize the blood DNA methylome of the giant panda, including its sequence context preferences and distribution across genomic functional elements; (2) identify seasonally differentially methylated regions (DMRs) and differentially methylated genes (DMGs) between winter and summer; and (3) reveal the biological processes and signaling pathways involved in seasonal methylation variation through Gene Ontology (GO) and Kyoto Encyclopedia of Genes and Genomes (KEGG) enrichment analyses, thereby elucidating the potential epigenetic regulatory strategies of giant pandas in response to seasonal environmental stressors. Our findings will provide novel molecular evidence for understanding seasonal adaptation in endangered mammals, and offer theoretical support and candidate molecular markers for the seasonal health management of captive giant pandas and the adaptive assessment of individuals for reintroduction.

## 2. Materials and Methods

### 2.1. Experimental Animals and Sample Collection

A total of 12 blood samples were collected from three healthy adult female giant pandas (codes: Q, H, and X) undergoing wild training under natural field conditions at the Sichuan Daxiangling Research Base of Giant Panda Reintroduction (29°32′50″–29°33′55″ N, 102°50′14″–102°51′03″ E; elevation 2410–2560 m), located within the Daxiangling Nature Reserve in southwestern China [10,11]. Sampling spanned three years (2023–2025): individual X was sampled in all three years, H in two years, and Q in one year, with one winter (W; mean temperature −5 °C) and one summer (S; mean temperature 15 °C) sample collected from each individual in each sampling year, yielding six winter–summer pairs (three for X, two for H, and one for Q). This study was approved by the Institutional Animal Care and Use Committee of the Chengdu Research Base of Giant Panda Breeding (Approval No. 2023017), and all sampling procedures were conducted in accordance with animal ethics regulations without anesthesia. Through the successful implementation of an artificially assisted soft-release training program [48], giant pandas were trained to voluntarily extend their forelimbs, thereby minimizing sampling-related stress [16,49]. Blood was drawn from the forelimb vein, anticoagulated with EDTA, and stored at −80 °C until use. Detailed sample information is presented in Table 1, with samples labeled according to the rule “individual + last two digits of year + season” (e.g., Q23W).

### 2.2. DNA Extraction and Library Construction

Genomic DNA was extracted using the Qiagen DNeasy Blood & Tissue Kit (Qiagen, Hilden, Germany). DNA integrity was assessed by agarose gel electrophoresis, and purity was assessed using a NanoDrop 2000 spectrophotometer (Thermo Fisher Scientific, Waltham, MA, USA), with OD260/280 values of 1.8–2.0. For each sample, 1.0 μg of DNA was spiked with 0.5% lambda DNA as a bisulfite conversion control, fragmented to 200–400 bp using a Covaris S220 ultrasonic crusher (Woburn, MA, USA), and treated with the EZ DNA Methylation-Gold Kit (Zymo Research, Irvine, CA, USA). Libraries were constructed using the Illumina TruSeq DNA Methylation Kit (Illumina, San Diego, CA, USA) and quality-checked on an AATI Fragment Analyzer (Advanced Analytical Technologies, Inc., Ames, IA, USA) and by qPCR The qualified libraries were sent to Novogene (Beijing, China) for paired-end sequencing (150 bp) on the Illumina NovaSeq 6000 platform (Illumina, San Diego, CA, USA).

### 2.3. Bioinformatic Analysis

#### 2.3.1. Quality Control

Raw reads were assessed using FastQC v0.11.8 and filtered with fastp v0.23.1 [50,51] with the following parameters: adapter sequences were removed; bases with quality < 3 or containing N at read ends were trimmed; reads were truncated using a 4 bp sliding window when the mean window quality dropped below 15; reads shorter than 36 bp after trimming and unpaired reads were discarded. Clean reads were re-validated with FastQC v0.11.8 and used for subsequent analyses.

#### 2.3.2. Detection and Analysis of Methylation Sites

Clean reads were aligned to the giant panda reference genome (ASM200744v2) using Bismark v0.24.0 [52] (Bowtie2-based). PCR duplicates were removed with deduplicate_bismark, and methylated cytosines were extracted with bismark_methylation_extractor. The bisulfite non-conversion rate (*r*) was estimated from C sites in the unmethylated lambda DNA spike-in. Cytosines with coverage ≥ 5× were retained, and methylation calling was performed by a binomial test based on methylated read counts (mC), total read counts (mC + umC), and the non-conversion rate, with FDR correction (*p* < 0.05). The CNSknowall platform (https://cnsknowall.com, last accessed on 17 September 2026), a comprehensive web service for data analysis and visualization, was utilized.

#### 2.3.3. DMR Analysis

Differentially methylated loci (DMLs) and differentially methylated regions (DMRs) were identified using DSS v2.12.0 [53,54]. DSS estimates the dispersion at each cytosine site using a dispersion-shrinkage approach and, based on the beta-binomial distribution, performs a Wald test at each locus between the winter and summer groups (H0: μi1 = μi2, i.e., equal methylation levels between groups) to obtain locus-level *p*-values. Smoothing was enabled for DMR calling (smoothing = TRUE; smoothing.span = 200 bp), and the DMR-calling criteria were as follows: locus-level *p*-value < 1 × 10^−5^ (*p*.threshold = 1 × 10^−5^); minimum DMR length ≥ 50 bp (minlen = 50); at least 3 cytosine sites per DMR (minCG = 3); more than 50% of the sites within a DMR being statistically significant (*p* < 1 × 10^−5^; pct.sig = 0.5); and merging of adjacent DMRs within 100 bp (dis.merge = 100). No additional threshold on the magnitude of methylation difference was imposed (delta = 0); false positives were instead controlled by the stringent statistical criteria described above. DMR identification was performed independently for the CG, CHG, and CHH sequence contexts.

DMRs were anchored to gene bodies (TSS to TES) or promoter regions (2 kb upstream of the TSS): a DMR overlapping either region by at least 1 bp was assigned the corresponding gene as an anchored gene, i.e., a differentially methylated gene (DMG). The DMG classification was binary; the number, size, and magnitude of methylation difference in the overlapping DMRs were not additionally weighted. Relevant data were visualized using the CNSknowall platform (https://cnsknowall.com, last accessed on 17 September 2026).

#### 2.3.4. GO/KEGG Enrichment Analysis

GO enrichment analysis of DMGs was performed using GOseq v1.26.0 [55], which corrects for gene length bias, with Benjamini–Hochberg (BH) adjustment for multiple testing (adjusted *p* < 0.05). KEGG pathway enrichment of the same DMG set was conducted using KOBAS v3.0 [56,57], also applying BH correction (adjusted *p* < 0.05). The bar chart and bubble chart of enrichment analysis were generated by SRplot (https://www.bioinformatics.com.cn, last accessed on 17 September 2026), an online platform for data analysis and visualization [58].

## 3. Results

### 3.1. Quality of WGBS Data

Sequencing data quality statistics are summarized in Table 2. After quality filtering with fastp v0.23.1, the 12 samples yielded 342–508 million clean reads each, with clean read retention rates above 96%, Q30 base quality scores above 96%, and GC contents ranging from 21.44% to 22.12%. Using lambda DNA as the spike-in control, bisulfite conversion rates ranged from 98.99% to 99.71%, comparable to those reported in previous giant panda blood WGBS studies (above 99.11%) [46]. These results indicate that the WGBS data in this study are of high quality and reliable, providing a solid foundation for downstream analyses such as DMR identification and functional enrichment.

### 3.2. Mapping to the Reference Genome

Clean reads were aligned to the giant panda reference genome (ASM200744v2) using Bismark v0.24.0. The total mapping rates ranged from 83.51% to 86.45%, and the unique mapping rates ranged from 81.21% to 83.99% across samples (Table 3), indicating satisfactory alignment performance [42,43,46]. In terms of genome-wide coverage, the average coverage depth ranged from 22.35× to 32.80×, and the proportion of genomic sites covered at ≥1× exceeded 95% in all samples (95.97–96.15%), consistent with the WGBS depth recommended by Ziller et al. for DMR detection [59].

### 3.3. Genome-Wide Methylation Characteristics

**Figure 1:**
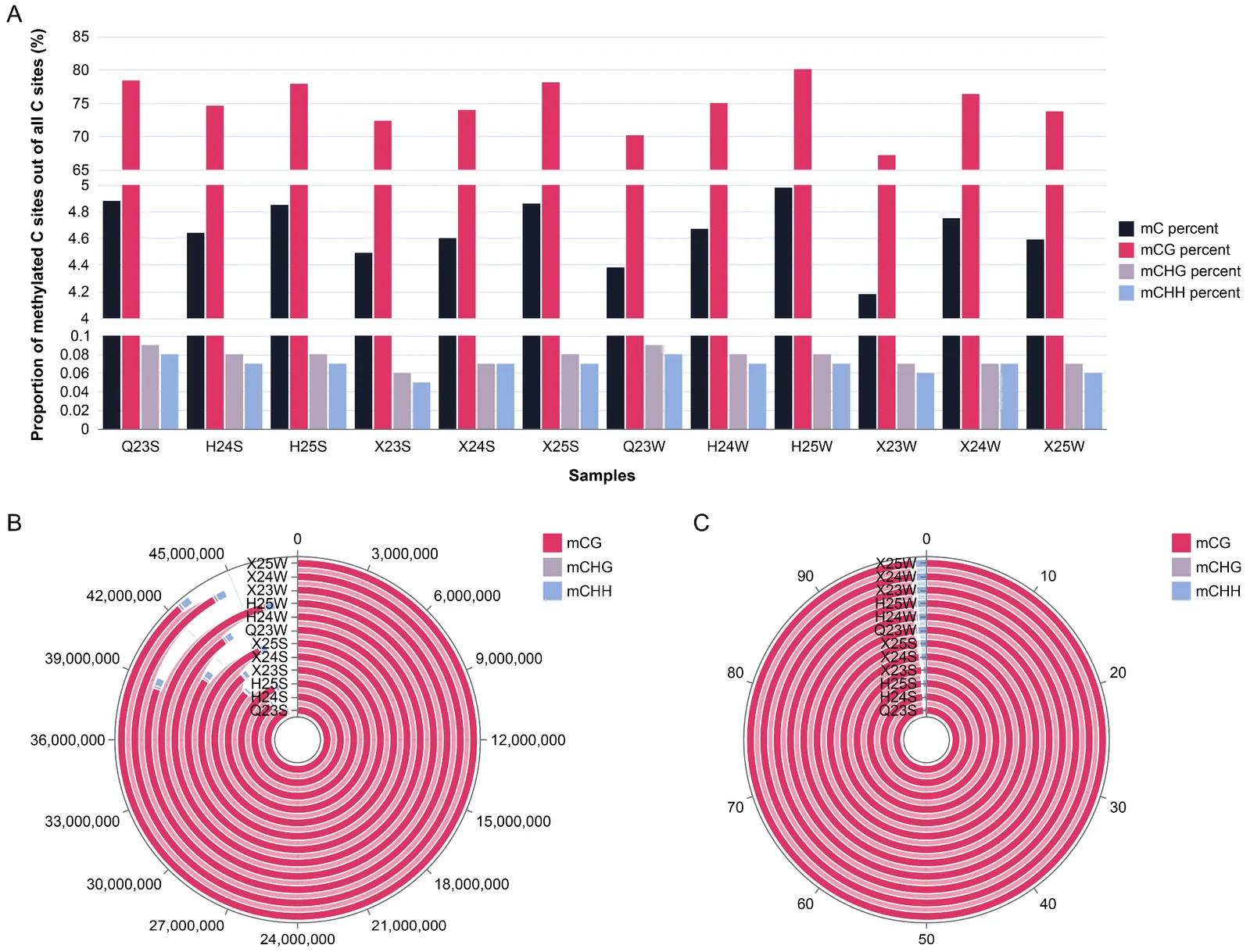
Statistics of methylated cytosine sites in each sample. (**A**) Proportion of methylated cytosine sites in each sequence context relative to all cytosine sites. (**B**) Number of methylated cytosine sites in each sequence context. (**C**) Proportion of methylated cytosine sites in each sequence context relative to total methylated cytosine sites. Total number of methylated cytosine sites in the sequencing data: mC = mCG + mCHG + mCHH. Proportion of methylated cytosine sites in the CG context: mCG% = (mCG/mC) × 100%. Proportion of methylated cytosine sites in the CHG context: mCHG% = (mCHG/mC) × 100%. Proportion of methylated cytosine sites in the CHH context: mCHH% = (mCHH/mC) × 100%.

Genome-wide methylation ratios (mC/C) ranged from 4.18% to 4.98% (Figure 1A). By sequence context, methylation ratios in the CG context (mCG/C) ranged from 67.19% to 80.08%, whereas those in the CHG and CHH contexts were both below 0.1% (Figure 1A). In terms of the composition of methylated C sites, mCG was overwhelmingly dominant, accounting for over 98.33% of all methylated C sites, with mCHG and mCHH representing only minor fractions (Figure 1B,C). This pattern was highly consistent across all 12 samples, with no obvious differences observed between seasons or among individuals. These results indicate that DNA methylation in the blood of wild-trained giant pandas occurs predominantly in the CG context; non-CG methylation, although detectable, is present at extremely low levels, consistent with previous giant panda methylation studies [43,45,46] and with the general characteristic of mammalian DNA methylation occurring mainly at CpG dinucleotides [18,20]. These results also suggest that the CG context should be the primary focus in subsequent DMR analyses, whereas data from the CHG and CHH contexts are relatively limited in statistical power.

**Figure 2:**
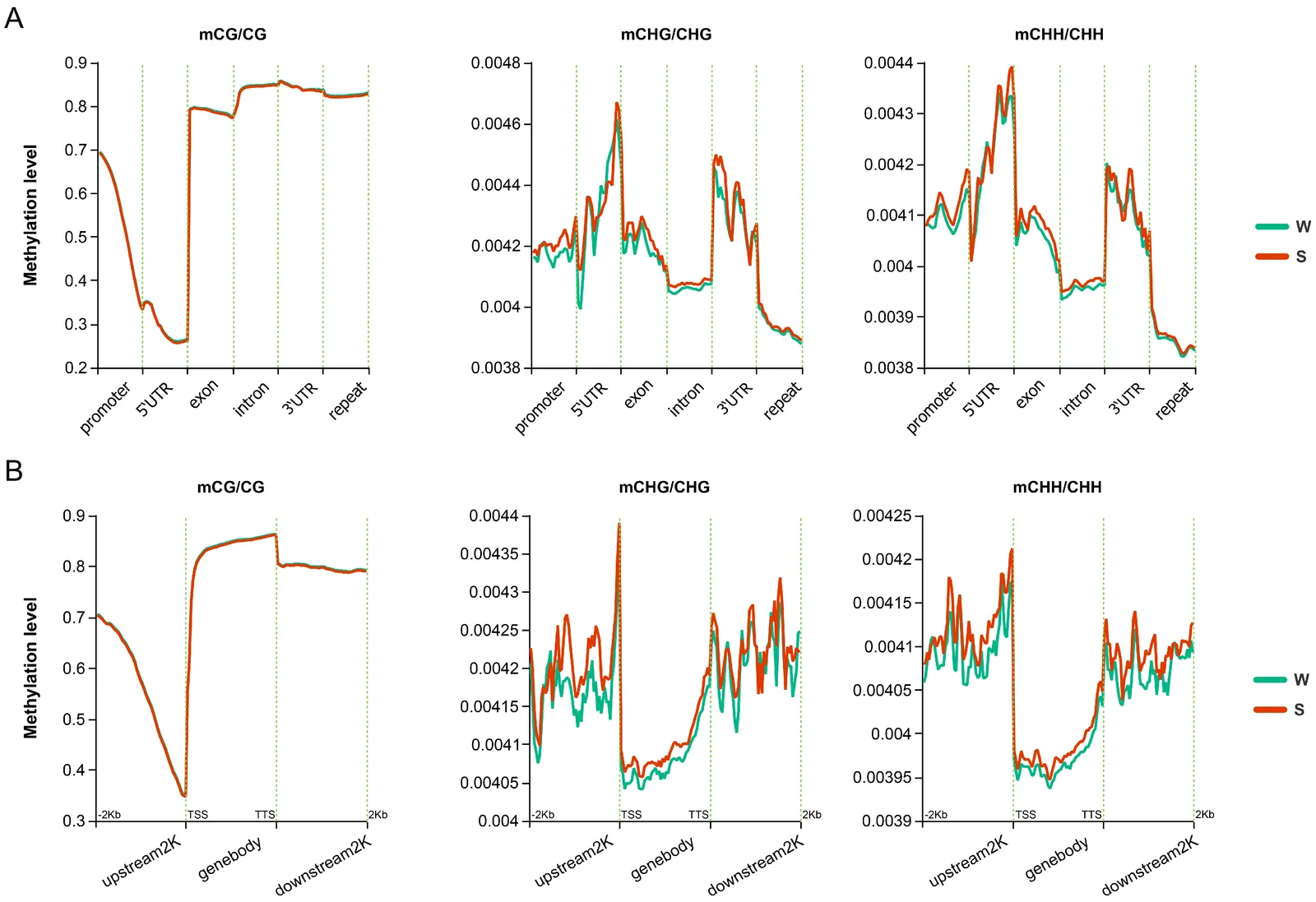
Distribution characteristics of methylation levels across genomic functional regions and gene body flanking regions in three sequence contexts. (**A**) Methylation level distribution across different genomic functional elements and repetitive sequence regions. (**B**) Methylation level distribution across genebody and upstream/downstream flanking regions.

By quantifying methylation levels across genomic functional elements and repeat regions (Figure 2A), we found that in the CG context, methylation levels were relatively high in introns, 3′ UTRs, repeat regions, and exons, followed by promoters, and lowest in 5′ UTRs; moreover, methylation levels in proximal promoter regions were lower than those in distal promoter regions. The distribution patterns in the CHG and CHH contexts were relatively similar, with relatively high levels in 5′ UTRs and 3′ UTRs, followed by promoters and exons, then introns, and lowest in repeat regions. No evident differences between the W and S groups were observed across these regions. Statistics for the genebody and its 2 kb flanking regions (Figure 2B) showed that in the CG context, methylation levels were lowest in the 2 kb region upstream of the TSS, gradually increased within the genebody, peaked near the TTS, and declined in the 2 kb region downstream of the TTS, displaying an overall pattern of “genebody–high, flanking–low”. In contrast, in the CHG and CHH contexts, methylation levels were markedly lower and exhibited the opposite pattern of “genebody–low, flanking–high”. Similarly, the distribution patterns of the W and S groups were highly similar in all three contexts, with no evident inter-group differences.

### 3.4. DMR Characteristics

**Figure 3:**
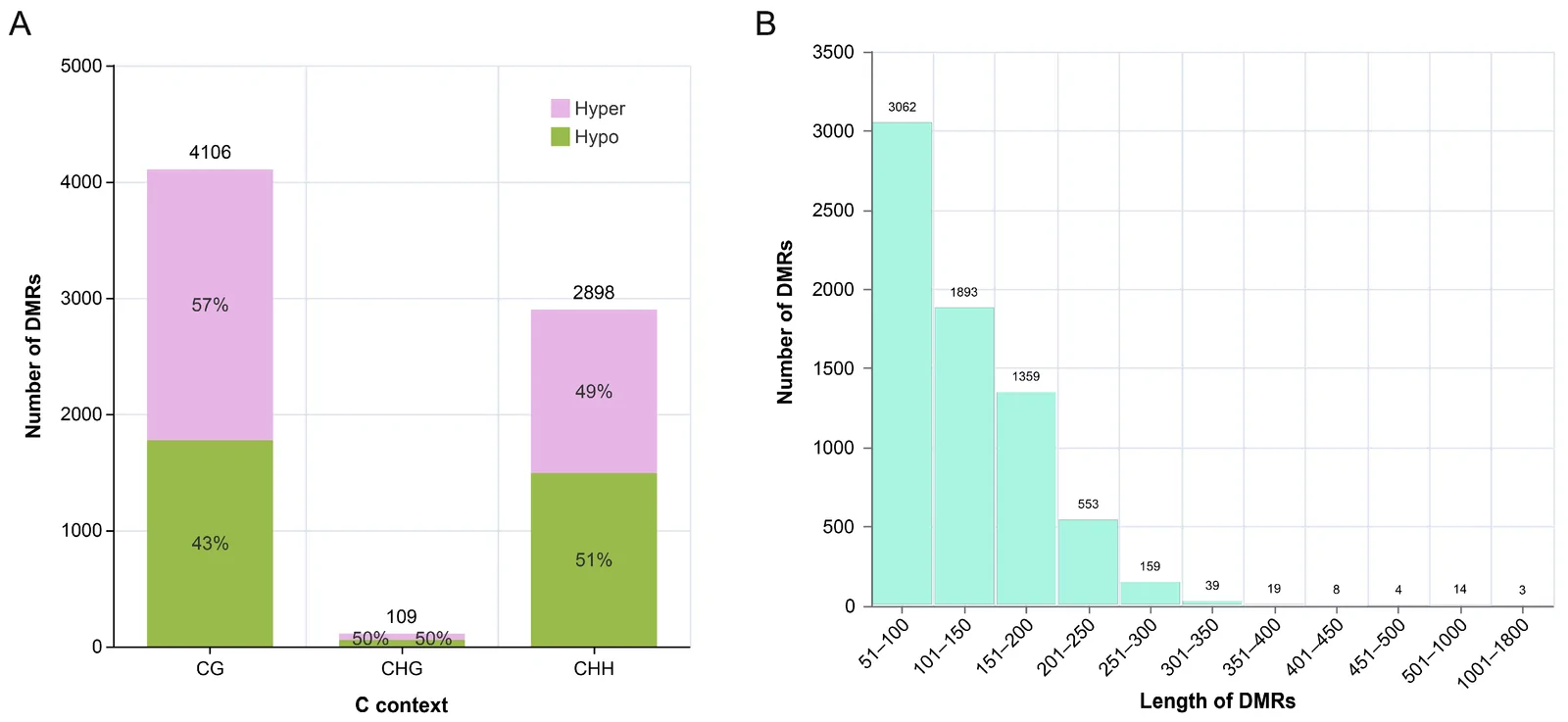
Statistics of the number and length of DMRs in the winter vs. summer comparison. (**A**) Number of DMRs in the three sequence contexts. (**B**) Distribution of DMR lengths (x-axis: DMR length intervals in bp; y-axis: number of DMRs in each length interval).

A total of 7113 DMRs were identified in the winter vs. summer comparison using DSS v2.12.0 (Figure 3A). Among these, CG-type DMRs were the most abundant (57.73%), with hypermethylation clearly predominant; CHH-type DMRs ranked second (40.74%), with slightly more hypomethylation; and CHG-type DMRs were the least frequent (1.53%), with a balanced distribution of hyper- and hypomethylation (Figure 3A). These results indicate that season-associated methylation changes in giant panda blood occur predominantly in the CG context.

Length analysis showed that the 7113 DMRs ranged from 51 to 1796 bp and were predominantly concentrated in the 51–200 bp range (6314 DMRs; 88.77%; Figure 3B). Specifically, DMRs of 51–100, 101–150, 151–200, 201–500, and >500 bp accounted for 43.05%, 26.61%, 19.11%, 10.99%, and 0.24%, respectively (Figure 3B). The number of DMRs decreased markedly with increasing length [46], suggesting that seasonal environmental variation primarily induces short DMRs (≤200 bp).

**Figure 4:**
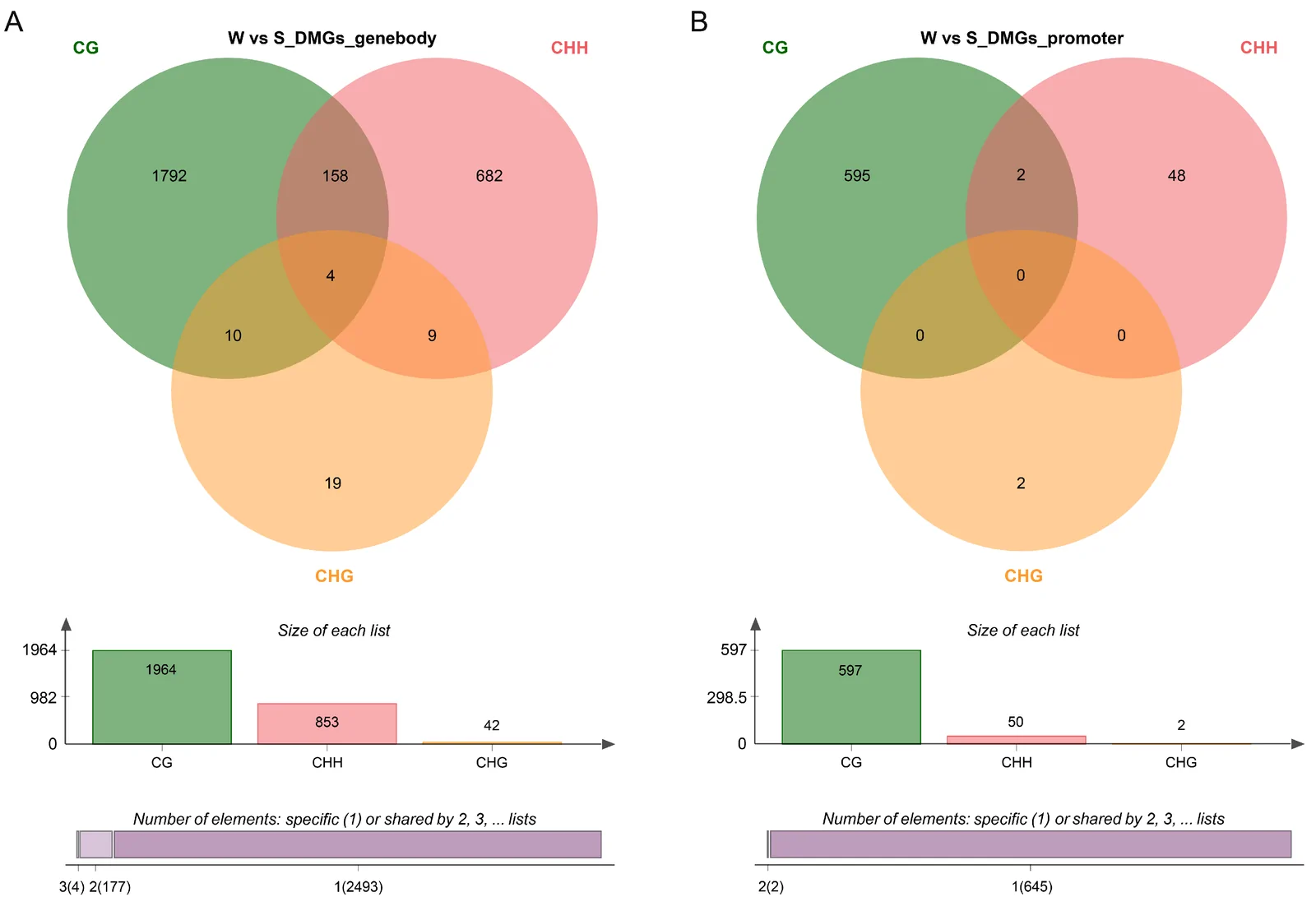
Venn diagrams of DMGs in three sequence contexts. (**A**) DMGs in genebody regions. (**B**) DMGs in promoter regions.

A total of 3063 DMGs associated with DMRs were identified through annotation. To assess the impact of sequence context on DMG classification, DMGs were anchored to genebody (TSS to TTS) and promoter regions separately. In genebody regions, 2674 DMGs were identified, with CG-type DMGs overwhelmingly predominant, followed by CHH and CHG types, with minimal overlap among the three contexts (Figure 4A). In promoters, CG-type DMGs likewise dominated, with CHH and CHG types being rare, and only 2 DMGs overlapping between the CG and CHH contexts (Figure 4B). Collectively, the number of DMGs in each context exceeded that in promoters, and CG-type DMGs dominated both regions, indicating that DMGs are primarily enriched within gene bodies and that the CG context should remain the primary focus [46].

### 3.5. GO Enrichment Analysis of DMGs

Since CHG- and CHH-type DMGs were few in number and showed no statistically significant enrichment (*p* ≥ 0.05), subsequent GO enrichment analysis was performed only on CG-type DMGs. To clarify the biological implications of methylation change direction, the gene sets were divided into all DMGs, winter-hypermethylated genes, and winter-hypomethylated genes for separate enrichment analysis (Tables S1–S3).

#### 3.5.1. Overall Functional Features of All DMGs

**Figure 5:**
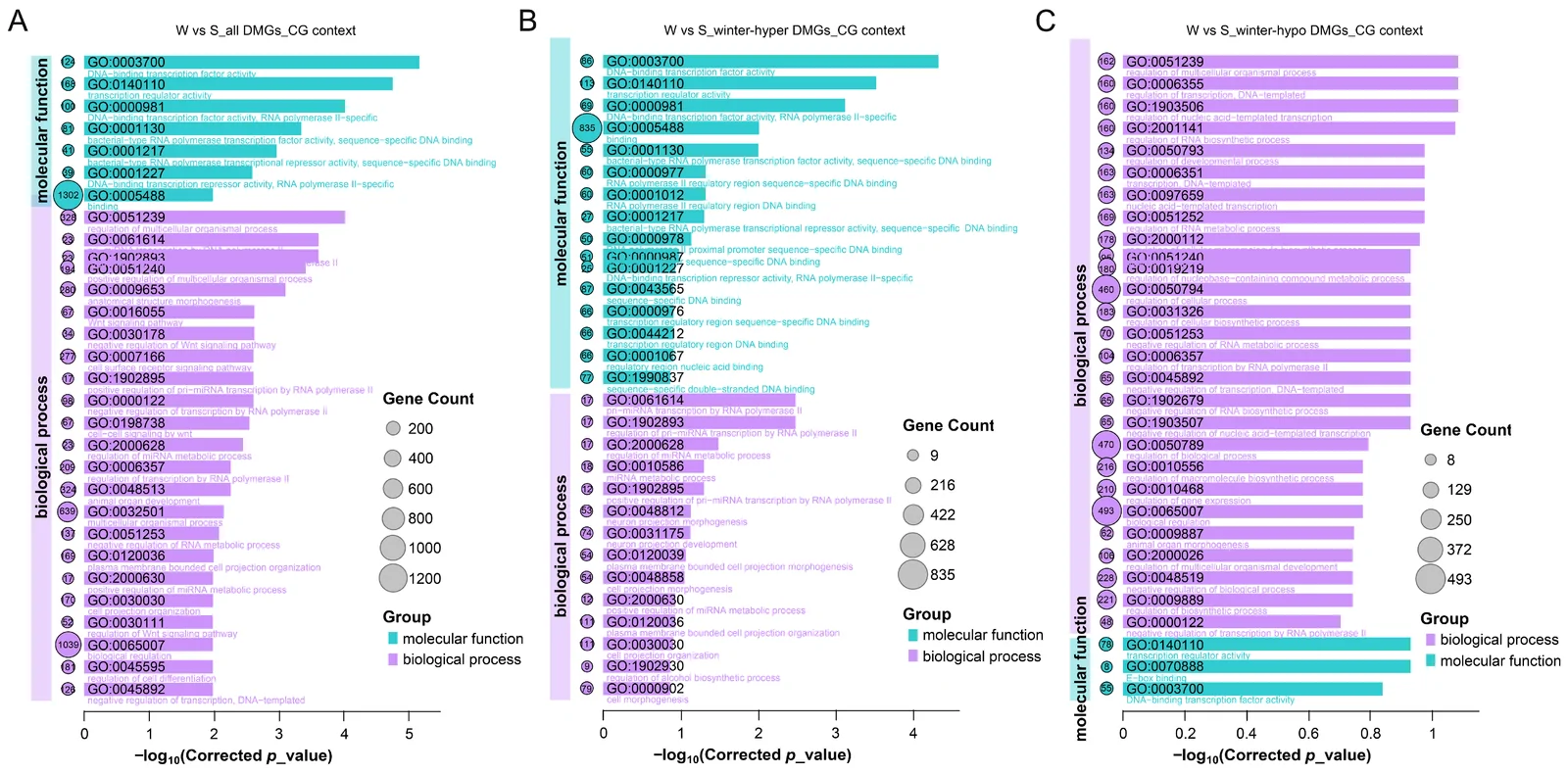
GO enrichment analyses of DMGs in the CG context. (**A**) All DMGs. (**B**) winter-hypermethylated genes. (**C**) winter-hypomethylated genes. Only the top 30 most enriched GO terms are shown.

A total of 81 significantly enriched terms (*p* < 0.05) were obtained among all CG-type DMGs, including 70 biological processes and 11 molecular functions (Table S1 and Figure 5A).

Transcription regulation-related terms were the most prominent among all DMGs. DNA-binding transcription factor activity (GO:0003700), transcription regulator activity (GO:0140110), DNA-binding transcription factor activity, RNA polymerase II-specific (GO:0000981), bacterial-type RNA polymerase transcription factor activity, sequence-specific DNA binding (GO: 0001130), and regulation of transcription, DNA-templated (GO:0006355) were all significantly enriched, indicating that DNA methylation is broadly involved in the reprogramming of transcriptional programs.

Developmental and morphogenesis-related processes were also significantly enriched, including anatomical structure morphogenesis (GO:0009653), animal organ development (GO:0048513), system development (GO:0048731), and multicellular organism development (GO:0007275), suggesting that methylation may influence seasonal tissue remodeling or cell turnover.

In terms of signal transduction and cell communication, the cell surface receptor signaling pathway (GO:0007166), signaling (GO:0023052), cell communication (GO:0007154), Wnt signaling pathway (GO:0016055), and negative regulation of Wnt signaling pathway (GO:0030178) were all significantly enriched, indicating that signaling pathways are important targets of methylation regulation.

miRNA metabolism-related terms were also significantly enriched at the overall level, including pri-miRNA transcription by RNA polymerase II (GO:0061614), regulation of pri-miRNA transcription by RNA polymerase II (GO:1902893), positive regulation of pri-miRNA transcription by RNA polymerase II (GO:1902895), and regulation of miRNA metabolic process (GO:2000628).

Notably, immune-related terms such as innate immune response (GO:0045087, *p* = 0.44) and immune system process (GO:0002376, *p* = 0.44) did not reach statistical significance.

#### 3.5.2. Specific Enrichment Features of Winter-Hypermethylated Genes

A total of 11 significantly enriched terms (*p* < 0.05) were obtained for the winter-hypermethylated gene set, including 8 molecular functions and 3 biological processes (Table S2 and Figure 5B).

For transcriptional regulation, DNA-binding transcription factor activity (GO:0003700) and transcription regulator activity (GO:0140110) were the most significant terms, and bacterial-type RNA polymerase transcriptional repressor activity (GO:0001217, *p* = 4.98 × 10^−2^) approached significance.

Regarding miRNA metabolism, pri-miRNA transcription by RNA polymerase II (GO:0061614), regulation of pri-miRNA transcription by RNA polymerase II (GO:1902893), and regulation of miRNA metabolic process (GO:2000628) were all significantly enriched.

Neuron development-related terms such as neuron projection morphogenesis (GO:0048812, *p* = 0.07), neuron projection development (GO:0031175, *p* = 0.08), cell morphogenesis involved in neuron differentiation (GO:0048667, *p* = 0.14), and axonogenesis (GO:0007409, *p* = 0.22) showed marginal enrichment trends.

#### 3.5.3. Specific Enrichment Features of Winter-Hypomethylated Genes

GO enrichment of the winter-hypomethylated gene set did not reach the significance threshold (*p* < 0.05), but several functional categories showed marginal enrichment trends (Table S3 and Figure 5C).

In transcriptional regulation, regulation of transcription, DNA-templated (GO:0006355, *p* = 0.08), regulation of RNA biosynthetic process (GO:2001141, *p* = 0.08), and regulation of nucleobase-containing compound metabolic process (GO:0019219, *p* = 0.12) showed enrichment trends.

In terms of immune defense, the Toll-like receptor signaling pathway (GO:0002224, *p* = 0.06), innate immune response-activating signal transduction (GO:0002758, *p* = 0.08), activation of innate immune response (GO:0002218, *p* = 0.12), and positive regulation of cytokine production (GO:0001819, *p* = 0.10) all showed marginal enrichment trends.

For cellular activities, cell development (GO:0048869, *p* = 0.08), cell differentiation (GO:0030154, *p* = 0.12), and cell migration (GO:0016477, *p* = 0.36) also showed enrichment trends. Cellular component terms such as cell leading edge (GO:0031252, *p* = 0.29) and actin filament bundle (GO:0032432, *p* = 0.48) did not reach significance.

### 3.6. KEGG Enrichment Analysis of DMGs

Building on the GO enrichment analysis, we further performed KEGG pathway enrichment analysis for CG-type DMGs to reveal the metabolic and signal transduction pathways involving differentially methylated genes. Consistent with the GO analysis strategy, gene sets were divided into all DMGs, winter-hypermethylated genes, and winter-hypomethylated genes for enrichment analysis, with an adjusted *p* < 0.05 as the significance threshold (Tables S4–S6).

#### 3.6.1. Pathway Enrichment Features of All DMGs

**Figure 6:**
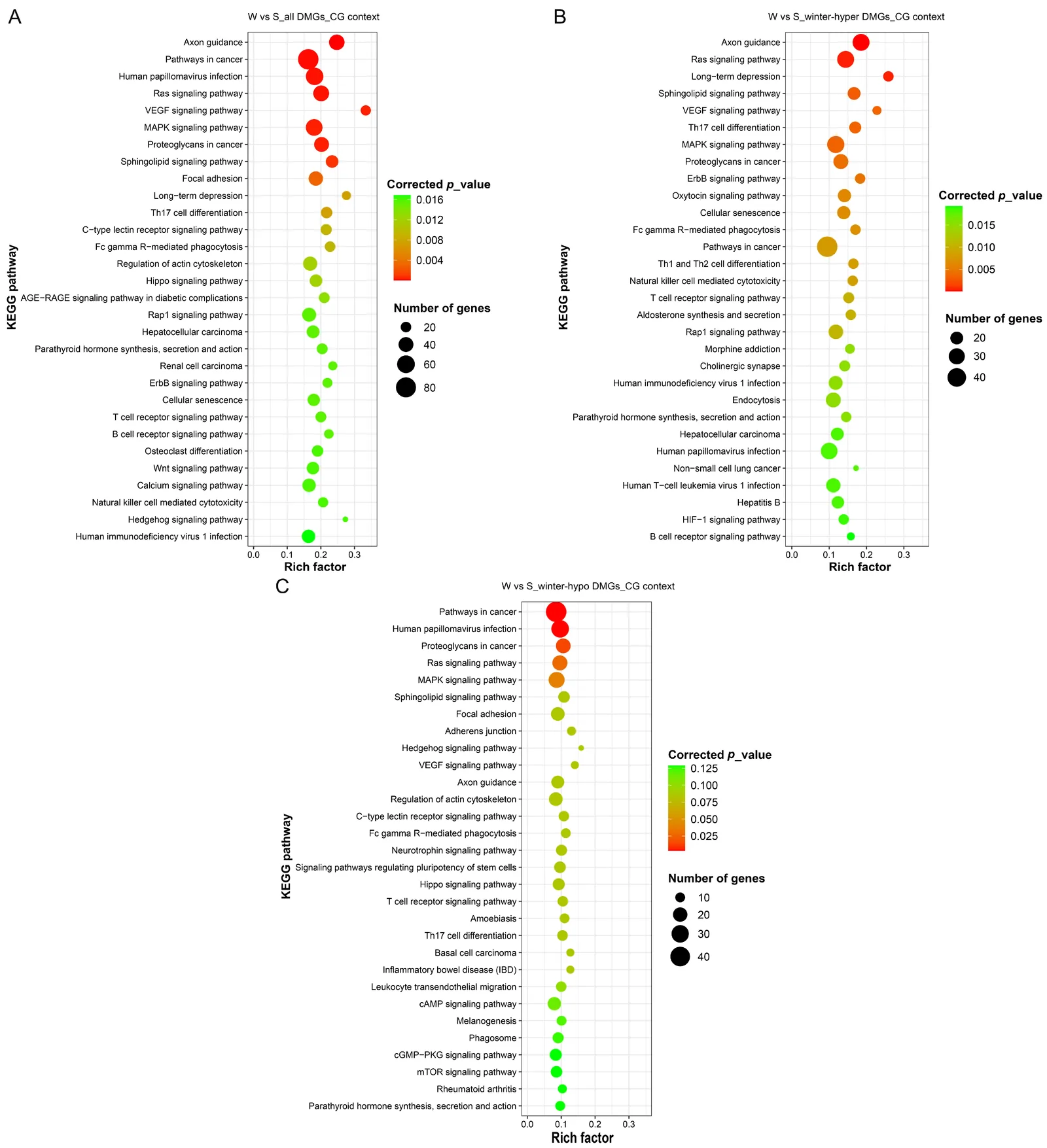
KEGG pathway enrichment analyses of DMGs in the CG context. (**A**) All DMGs. (**B**) winter-hypermethylated genes. (**C**) winter-hypomethylated genes. Only the top 30 most enriched pathways are shown.

A total of 56 significantly enriched KEGG pathways (*p* < 0.05) were identified among all DMGs, with functions mainly concentrated in categories such as signal transduction, cellular processes, and the immune system (Table S4 and Figure 6A).

In signal transduction, axon guidance (aml04360) was the most significantly enriched pathway, followed by Ras signaling pathway (aml04014), MAPK signaling pathway (aml04010), VEGF signaling pathway (aml04370), Rap1 signaling pathway (aml04015), cAMP signaling pathway (aml04024), calcium signaling pathway (aml04020), long-term depression (aml04730), and long-term potentiation (aml04720). In addition, Wnt signaling pathway (aml04310), Hippo signaling pathway (aml04390), and Hedgehog signaling pathway (aml04340) were also significantly enriched.

Regarding cellular processes, focal adhesion (aml04510), regulation of actin cytoskeleton (aml04810), and endocytosis (aml04144) were significantly enriched, while adherens junction (aml04520, *p* = 0.07) and tight junction (aml04530, *p* = 0.07) showed marginal enrichment trends. The signaling pathways regulating pluripotency of stem cells (aml04550) were also significantly enriched.

In immune-related pathways, Th17 cell differentiation (aml04659), C-type lectin receptor signaling pathway (aml04625), Fc gamma R-mediated phagocytosis (aml04666), T cell receptor signaling pathway (aml04660), B cell receptor signaling pathway (aml04662), osteoclast differentiation (aml04380), natural killer cell mediated cytotoxicity (aml04650), and leukocyte transendothelial migration (aml04670) were all significantly enriched.

In addition, disease-related pathways such as pathways in cancer (aml05200), human papillomavirus infection (aml05165), proteoglycans in cancer (aml05205), and hepatocellular carcinoma (aml05225) were also significantly enriched.

#### 3.6.2. KEGG Pathway Features of Winter-Hypermethylated Genes

The winter-hypermethylated gene set yielded 56 significantly enriched KEGG pathways (*p* < 0.05) (Table S5 and Figure 6B).

In signal transduction, axon guidance (aml04360) was the most significantly enriched pathway, while the Ras signaling pathway (aml04014), MAPK signaling pathway (aml04010), VEGF signaling pathway (aml04370), Rap1 signaling pathway (aml04015), calcium signaling pathway (aml04020), and cGMP-PKG signaling pathway (aml04022) were significantly enriched, and the cAMP signaling pathway (aml04024, *p* = 0.06) showed a marginal enrichment trend.

In immune-related pathways, Th17 cell differentiation (aml04659), Th1 and Th2 cell differentiation (aml04658), T cell receptor signaling pathway (aml04660), B cell receptor signaling pathway (aml04662), natural killer cell mediated cytotoxicity (aml04650), Fc gamma R-mediated phagocytosis (aml04666), C-type lectin receptor signaling pathway (aml04625), and osteoclast differentiation (aml04380) were all significantly enriched.

Regarding cell junctions and cytoskeletal regulation, regulation of actin cytoskeleton (aml04810) and focal adhesion (aml04510) were significantly enriched, whereas tight junction (aml04530, *p* = 0.17) and adherens junction (aml04520, *p* = 0.18) showed enrichment trends.

#### 3.6.3. Pathway Enrichment Features of Winter-Hypomethylated Genes

KEGG enrichment analysis of the winter-hypomethylated gene set yielded only 5 significantly enriched pathways (*p* < 0.05) (Table S6 and Figure 6C). In signal transduction, the MAPK signaling pathway (aml04010) and Ras signaling pathway (aml04014) were significantly enriched. In addition, disease-related pathways such as pathways in cancer (aml05200), human papillomavirus infection (aml05165), and proteoglycans in cancer (aml05205) were significantly enriched among hypomethylated genes.

## 4. Discussion

### 4.1. Data Quality and Genome-Wide Methylation Characteristics

WGBS is widely regarded as the gold standard for DNA methylation profiling and has been widely used [41,46,60,61]. The WGBS data in this study had an average coverage depth of 22.35×–32.80× and a genome coverage of >95%, meeting the depth requirements for DMR detection proposed by Ziller et al. [59]. That study demonstrated that a coverage depth of approximately 30× is sufficient for high-sensitivity genome-wide DMR identification, while further increases in per-sample sequencing depth yield limited improvement in DMR discovery; under a fixed total sequencing budget, increasing the number of biological replicates enhances statistical power more effectively than simply increasing sequencing depth [59]. In this study, six biological replicates were set per group, combined with sufficient sequencing depth, providing a reliable data foundation for subsequent differential methylation analysis.

Regarding genome-wide methylation characteristics, the genome-wide methylation level (mC/C) was 4.18–4.98%, CG-context methylation was 67.19–80.08%, while CHG and CHH levels were both below 0.1%, and mCG accounted for over 98.33% of all methylated cytosines. This pattern was highly consistent across all 12 samples, with no evident seasonal or individual variation, consistent with previous giant panda methylation studies [43,45,46] and with the fundamental feature of mammalian DNA methylation occurring predominantly at CG dinucleotides [17,18,20,61]. Regarding the DMRs, CG-, CHH-, and CHG-type DMRs accounted for 57.73% (4106), 40.74% (2898), and only 1.53% (109), respectively, further confirming the central role of the CG context in responses to seasonal environmental change. DMR lengths were predominantly concentrated within 51–200 bp (88.77%), a short-fragment DMR distribution consistent with that reported in a previous giant panda blood WGBS study by Jie et al. [46]. Short DMRs typically correspond to transcription factor binding sites or local regulatory elements, suggesting that seasonal temperature variation primarily affects fine-scale regulatory regions rather than large-scale chromatin domains. Regarding functional-region distribution, CG-type DMGs dominated both gene bodies (1964) and promoters (597), and the number of DMGs of each type in gene bodies exceeded that in promoters, indicating that seasonal temperature variation primarily influences gene expression through CG-context methylation modifications within gene bodies [19].

### 4.2. “Summer Immune Enhancement, Winter Energy Conservation”: Epigenetic Responses of Giant Pandas to Seasonal Multi-Factor Environments

Notably, samples in this study were collected from natural field habitats. The methylation differences between winter and summer samples should therefore be interpreted as an integrated epigenetic response to multiple co-occurring environmental factors—including temperature, photoperiod, food resources, and pathogen pressure—rather than as independent effects of any single factor. Accordingly, the discussions of specific pathways or genes below are directional inferences that should be understood within the overall framework of natural seasonal dynamics. This “real-world, multi-factor coupling” characteristic represents the core value distinguishing this study from controlled experiments.

Within this multi-factor framework, previous studies have revealed patterns of DNA methylation responses to seasonal environmental stress from different perspectives. Regarding temperature, cold stress in zebrafish affects methylation of genes involved in antioxidation, chromatin modification, and immune processes [34]; European sea bass exhibit developmental-stage-dependent methylation remodeling under only a 2 °C temperature increase [35]; and in mammals, cold exposure can even transmit methylation changes across generations through the germline [29]. Together, these findings indicate that temperature is one of the core drivers of seasonal methylation variation. Regarding photoperiod, Stevenson and Prendergast first demonstrated in Siberian hamsters that short-day exposure induces seasonal physiological transitions by suppressing hypothalamic DNA methyltransferase expression and reducing *dio3* promoter methylation, a process reversible in spring [37]; sheep *MTNR1A* promoter methylation has also been shown to respond significantly to photoperiod changes [38], suggesting that photoperiod can function as a seasonal signal relatively independent of temperature. Regarding pathogen pressure, Lundregan et al. used RRBS to demonstrate in wild house sparrows that nematode infection induces differential methylation of genes related to innate and adaptive immunity [62], extending immune methylation regulation from controlled infection models to natural host–pathogen interaction contexts. Regarding food and energy, thirteen-lined ground squirrels exhibit specific methylation modifications of metabolism-related genes during natural hibernation with low metabolic rates [33], and seasonal methylation variation in wild birds is also closely associated with reproductive timing—a key node of resource allocation [4,39].

Collectively, these findings demonstrate that DNA methylation serves as a sensitive integrative mediator of seasonal multi-factor environments, with conservation across evolution from fish to mammals [4,24,25,29,31,33,34,35,37,38,39,41,62]. The life history of giant pandas provides a unique biological framework for understanding this integrative role: in summer, high temperatures and rainfall coincide with heightened tick and other pathogen activity [10,11], elevating immune defense demands; in winter, low temperatures, limited food resources, and increased foraging difficulty [8] elevate energy conservation demands. Within this ecological context, plastic changes in DNA methylation are considered a key mechanism for coping with seasonal energy and survival pressures [4,39]. The following sections discuss methylation evidence along two main themes: summer immune defense and winter energy conservation.

#### 4.2.1. Summer Immune Defense Module

Among winter-hypermethylated genes, immune-related pathways were significantly enriched, including Th17 cell differentiation (aml04659), T cell receptor signaling pathway (aml04660), B cell receptor signaling pathway (aml04662), natural killer cell-mediated cytotoxicity (aml04650), Fc gamma R-mediated phagocytosis (aml04666), and C-type lectin receptor signaling pathway (aml04625). This indicates that these immune pathways were relatively hypermethylated in winter and relatively hypomethylated in summer. This regulatory direction aligns closely with the field life history of giant pandas—increased tick and other pathogen pressure in summer activates immune pathways through hypomethylation to enhance defense, whereas reduced pathogen pressure in winter suppresses these pathways through hypermethylation to conserve energy.

Lundregan et al. found that house sparrows infected with parasites exhibited a slight reduction in overall DNA methylation, and candidate genes related to initial immune responses, innate and adaptive immune activation, and mucosal integrity showed differential methylation between infected and control groups [62]. Bind et al. demonstrated in human blood that ambient temperature and relative humidity directly modulate methylation levels of multiple immune-related genes, including Toll-like receptor 2 (*TLR-2*) [32], indicating that temperature-mediated regulation of immune gene methylation is conserved in mammals. DNA methylation has been shown to play a decisive role in differentiation, maturation, and functional maintenance of immune cells, with T cell and B cell functions highly dependent on dynamic changes in their methylation profiles [63]. Jie et al. similarly found that DNA methylation participates in immune adaptation regulation in a blood WGBS study of wild-trained giant pandas [46], and Huang et al. reported in an age-related blood methylation study that expression of immune-related genes was negatively correlated with their methylation levels [45]. This study extends immune methylation regulation to the dimension of seasonal environmental adaptation, and the direction of methylation changes in immune pathways aligns with those reported in the above studies across different physiological states. In addition, Th1 and Th2 cell differentiation (aml04658, *p* = 0.01) was also significantly enriched among winter-hypermethylated genes, suggesting that T cell polarization toward different helper subtypes may undergo overall remodeling in summer to enhance immune responses against diverse intracellular and extracellular pathogens.

Notably, in the GO enrichment analysis of all DMGs, immune-related terms did not reach statistical significance (e.g., innate immune response, GO:0045087, *p* = 0.44), whereas immune pathways were significantly enriched at the KEGG level. This incomplete concordance between GO and KEGG results may reflect the synergistic nature of immune regulation—immune responses depend on coordinated changes of multiple genes within specific signaling cascades, a feature more readily detected by pathway-interaction-based KEGG statistical tests than by gene-term classification-based GO tests [64].

#### 4.2.2. Winter Energy Conservation Module

Winter-hypermethylated genes were simultaneously significantly enriched in multiple energy-consuming pathways, suggesting that winter systematically suppresses high-energy biological processes through methylation modifications. At the transcriptional regulation level, DNA-binding transcription factor activity (GO:0003700) and transcription regulator activity (GO:0140110) were the most significant GO terms among winter-hypermethylated genes, suggesting that winter suppresses transcription factor expression or activity through methylation, globally reducing transcriptional output. Transcription is one of the most energy-consuming cellular processes, and protein synthesis and associated transcriptional processes account for a substantial fraction of total cellular ATP consumption [65]; suppression of these processes contributes to energy conservation.

For signal transduction, the MAPK signaling pathway (aml04010), Ras signaling pathway (aml04014), and calcium signaling pathway (aml04020) were all significantly enriched. The MAPK cascade transmits signals through multistep phosphorylation/dephosphorylation reactions accompanied by continuous ATP hydrolysis, and maintenance of pathway activity has measurable effects on cellular energy status [66,67]; Ras proteins, as small GTPases, function as molecular switches through cyclic GTP/GDP binding-state transitions, and their activity maintenance likewise depends on continuous GTP hydrolysis [68]; the calcium signaling pathway relies on Ca^2+^-ATPase to actively transport Ca^2+^ against concentration gradients, representing a typical high-ATP-consuming cellular process [69]. These phosphorylation cascades, GTPase cycles, and active ion transport all constitute high-energy biological processes, and winter suppression through methylation can synergistically reduce the overall energy cost of signal transduction.

The cAMP signaling pathway (aml04024, *p* = 0.06) also showed an enrichment trend; this pathway broadly regulates metabolism and gene expression [70], with its synthesis and downstream phosphorylation both using ATP as a substrate [71,72]. Winter suppression of this pathway through methylation reduces ATP expenditure for signal generation and transmission, contributing to overall energy conservation.

For the cytoskeleton and cell migration, regulation of actin cytoskeleton (aml04810) and focal adhesion (aml04510) was significantly enriched. Actin polymerization/depolymerization is accompanied by continuous ATP hydrolysis on F-actin [73], representing one of the high-energy cellular processes; winter suppression of cytoskeletal remodeling reduces the corresponding energy expenditure.

For development and growth, the cell cycle pathway (aml04110) showed an enrichment trend among winter-hypermethylated genes; cell division is one of the most energy-consuming cellular processes, and its suppression helps suspend growth and conserve resources. The Hippo signaling pathway (aml04390, *p* = 0.06) also showed an enrichment trend, as this pathway suppresses cell proliferation and organ growth while promoting cellular quiescence. In addition, the Wnt signaling pathway (aml04310, *p* = 0.07) and Hedgehog signaling pathway (aml04340, *p* = 0.14) showed enrichment trends among hypermethylated genes, mutually corroborating the enrichment of “negative regulation of Wnt signaling pathway” (GO:0030178) in the GO analysis, indicating that suppression of developmental signaling constitutes an important component of the winter energy-conservation strategy.

A study of winter sperm methylation in Angus cattle similarly found that cold exposure induces differential methylation of numerous genes related to metabolism, cytoskeleton, and signal transduction [31], consistent with the “winter suppression of high-energy pathways” pattern observed in blood in this study. Furthermore, similar methylation alterations of metabolism-related genes during hibernation have been observed in hibernating mammals such as the thirteen-lined ground squirrel [33]. These findings suggest that cold-induced “energy-conserving” methylation reprogramming may exhibit some degree of conservation across species, and that blood methylation may serve as an effective indicator of whole-body energy status.

#### 4.2.3. Potential Functions of Winter-Hypomethylated Genes

Winter-hypomethylated genes were significantly enriched in the MAPK signaling pathway (aml04010), Ras signaling pathway (aml04014), and disease-related pathways such as pathways in cancer (aml05200). Compared with the substantial enrichment of the hypermethylated gene set (56 pathways), the hypomethylated gene set yielded only 5 significantly enriched pathways, suggesting that active regulation during seasonal adaptation is primarily manifested as hypermethylation-mediated gene suppression rather than hypomethylation-mediated gene activation. This pattern aligns with the “global transcriptional repression” observed in hibernating animals—where epigenetic modifications actively suppress high-energy processes rather than actively activating specific metabolic pathways [33].

### 4.3. Limitations and Future Perspectives

This study has certain limitations, the potential impacts of which on the interpretation of the findings are clarified below.

First, the sample size consisted of only three individuals; although six samples were collected in both winter and summer, the limited number of individuals constrains a comprehensive assessment of inter-individual variation. Importantly, because each winter–summer pair was collected from the same individual within the same year, the seasonal comparisons inherently control for inter-individual genetic background and stable individual traits, and the reported seasonal DMRs are therefore unlikely to result from confounding by individual differences. Nevertheless, individual-specific seasonal responses may have contributed to part of the observed variation; this limitation introduces uncertainty—rather than systematic bias—into the estimates of seasonal methylation differences, while restricting the generalizability of our conclusions to the broader giant panda population.

Second, this study only analyzed the DNA methylation layer without concurrent transcriptome sequencing; therefore, causal relationships between methylation changes and gene expression alterations could not be directly established. This limitation does not affect the identification of DMRs and DMGs, which was based on genome-wide methylation data alone, but it introduces uncertainty into the functional interpretation: the roles of the DMGs were inferred from pathway-level enrichment, and the direction (activating or repressive) of the methylation changes could not be determined. The present findings should therefore be interpreted as hypothesis-generating.

In addition, owing to the strictly limited blood volume collectable from giant pandas, hematological parameters were unavailable and blood cell-type composition could not be assessed, which may represent an additional source of uncertainty in the methylation profiles.

Future studies should integrate RNA-seq data to systematically elucidate the complete molecular pathway linking temperature variation, DNA methylation alterations, gene expression regulation, and physiological functional adaptation, and should include larger cohorts to validate and extend our findings.

## 5. Conclusions

This study provides the first genome-wide evidence that blood DNA methylation mediates seasonal adaptation in the giant panda, highlighting epigenetic regulation as an important mechanism by which endangered mammals respond to seasonal environmental variation. Using WGBS, we systematically characterized the response patterns of giant panda blood DNA methylation to seasonal temperature variation. The results demonstrate that giant panda blood DNA methylation undergoes extensive reprogramming under seasonal environmental changes, exhibiting a differentiated regulatory pattern of “summer immune enhancement, winter energy conservation”: winter-hypermethylated genes are enriched in immune defense-related pathways, presumably preparing for summer tick and other pathogen pressures; meanwhile, winter-hypermethylated genes are also enriched in high-energy pathways such as transcriptional regulation, signal transduction, and cytoskeletal reorganization, suppressing these processes through methylation to reduce winter energy expenditure. This finding aligns closely with the direction of seasonal environmental pressures on wild giant pandas, providing new insights into the epigenetic adaptation of endangered species, as well as potential molecular markers and theoretical references for seasonal health management of captive giant pandas and adaptability assessment of individuals released into the wild.

## Figures and Tables

**Table 1:** Sample information overview.

Samples ID	Panda Individuals	Sampling Date	Sampling Season	Season Code	Feeding Situation	Age at Sampling
Q23W	Q	17 Feb. 2023	winter	W	culms and leaves	9 y/o
Q23S	Q	14 Jul. 2023	summer	S	leaves	9 y/o
H24W	H	24 Jan. 2024	winter	W	culms and leaves	7 y/o
H24S	H	13 Aug. 2024	summer	S	leaves	8 y/o
H25W	H	28 Dec. 2024	winter	W	culms and leaves	8 y/o
H25S	H	21 Aug. 2025	summer	S	leaves	9 y/o
X23W	X	17 Feb. 2023	winter	W	culms and leaves	6 y/o
X23S	X	14 Jul. 2023	summer	S	leaves	7 y/o
X24W	X	24 Jan. 2024	winter	W	culms and leaves	7 y/o
X24S	X	13 Aug. 2024	summer	S	leaves	8 y/o
X25W	X	29 Dec. 2024	winter	W	culms and leaves	8 y/o
X25S	X	7 Aug. 2025	summer	S	leaves	9 y/o

**Table 2:** WGBS data quality assessment.

Samples ID	Raw Reads	Raw Bases (Gb)	Clean Reads	Clean Reads (Gb)	Clean Reads Ratio (%)	Q30 (%)	GC (%)	BS Conversion Rate (%)
Q23S	480852126	144.26	463470195	127.67	96.39	96.63	21.77	99.70
H24S	405900463	121.77	391611278	107.86	96.48	96.63	21.66	99.70
H25S	475419617	142.63	458998708	126.45	96.55	96.65	21.74	99.70
X23S	363184859	108.96	350665857	96.55	96.55	96.70	21.99	98.99
X24S	457897711	137.37	440174461	121.23	96.13	96.45	21.44	99.70
X25S	458229664	137.47	441186916	121.46	96.28	96.69	21.98	99.69
Q23W	402402714	120.72	386838791	106.51	96.13	96.48	21.46	99.70
H24W	419104667	125.73	405209832	111.63	96.68	96.71	21.64	99.70
H25W	525994971	157.80	507882094	139.94	96.56	96.70	21.81	99.70
X23W	354227966	106.27	341753825	94.10	96.48	96.65	21.69	99.69
X24W	443692331	133.11	428557127	118.10	96.59	96.72	21.64	99.71
X25W	369977097	110.99	358163471	98.65	96.81	96.77	22.12	99.69
Q23S	480852126	144.26	463470195	127.67	96.39	96.63	21.77	99.70

**Table 3:** Alignment statistics of clean reads to the reference genome.

Samples ID	Total Reads	Mapped Reads	Mapping Rate (%)	Unique Mapping Rate (%)	Sites_CovgMean (×)	Sites_NumCovg1 (%)
Q23S	463470195	398146603	85.91	83.47	31.22	96.09
H24S	391611278	337133699	86.09	83.67	26.46	96.03
H25S	458998708	395871619	86.25	83.79	30.19	96.09
X23S	350665857	294149772	83.88	81.46	25.04	95.97
X24S	440174461	379081415	86.12	83.73	28.88	96.04
X25S	441186916	370436503	83.96	81.50	31.70	96.08
Q23W	386838791	331034349	85.57	83.25	24.39	96.04
H24W	405209832	349621621	86.28	83.88	26.78	96.04
H25W	507882094	438210505	86.28	83.85	32.80	96.15
X23W	341753825	285396744	83.51	81.21	22.35	95.98
X24W	428557127	370490891	86.45	83.99	29.22	96.03
X25W	358163471	300324510	83.85	81.43	25.27	96.01
Q23S	463470195	398146603	85.91	83.47	31.22	96.09

## Data Availability

The data presented in this study are available on request from the corresponding author due to ethical and conservation restrictions concerning the endangered giant panda.

## Supplementary Materials

The following supporting information can be downloaded at https://www.mdpi.com/article/10.3390/ani16203286/s1. Table S1: GO enrichment results of all DMGs in winter vs. summer group; Table S2: GO enrichment results of hypermethylated genes in winter vs. summer group; Table S3: GO enrichment results of hypomethylated genes in winter vs. summer group; Table S4: KEGG enrichment results of all DMGs in winter vs. summer group; Table S5: KEGG enrichment results of hypermethylated genes in winter vs. summer group; Table S6: KEGG enrichment results of hypomethylated genes in winter vs. summer group.
